# Thymoglobulin Induction Dosing Strategies in a Low-Risk Kidney Transplant Population: Three or Four Days?

**DOI:** 10.1155/2010/957549

**Published:** 2010-11-07

**Authors:** Karen L. Hardinger, Rafia S. Rasu, Rebecca Skelton, Brent W. Miller, Daniel C. Brennan

**Affiliations:** ^1^Division of Pharmacy Practice and Administration, University of Missouri-Kansas City, Kansas City, MO 64108, USA; ^2^Department of Internal Medicine, Washington University School of Medicine, 660 S. Euclid Avenue Campus Box 8126, St. Louis, MO 63110, USA

## Abstract

The optimal dose and duration of rabbit antithymocyte globulin (rATG) induction has not been defined. *Methods*. We compared the safety and efficacy of 2 dosing strategies, rATG 1.5 mg/kg for 4 days (*n* = 59) versus 2 mg/kg for 3 days (*n* = 59), in a retrospective, cohort study. *Results*. Two-year rejection-free survival was 95% in each group (*P* = .983). Renal function and infection rates were similar. The incidence of leucopenia was similar, although the 2 mg/kg group was more likely to be thrombocytopenic on day 2 (4% versus 28%, *P* = .04). Length of stay tended to be longer for the 1.5 mg/kg group (6.0 ± 3.7 versus 5.1 ± 1.9 days *P* = .104). A cost savings of $920 per patient for rATG were seen in the 2 mg/kg group (*P* = .122). *Conclusions*. Shorter, more intense dosing of rATG is safe and effective. The 3-day dose strategy resulted in a clinically shorter length of stay and may result in cost savings.

## 1. Introduction

Induction therapy, using potent immunosuppressive agents in the critical, early period of allograft placement with the goal of decreasing the risk of acute rejection and potentially allowing lower overall intensity of the maintenance immunosuppressive regimen, is common in kidney transplantation. The induction agent of choice, along with dose and duration is controversial, center-specific, and often based on limited clinical data. 

Rabbit antithymocyte globulin (rATG, Thymoglobulin, Genzyme, Cambridge, MA) is FDA approved for treatment of acute rejection at a dose of 1.5 mg/kg for 7–14 days based on the results of a multicenter, double-blind randomized trial [1, 2]. Although rATG is not currently FDA approved as induction therapy in kidney transplantation, it is the most commonly administered agent for this purpose. Over one-half of the 70% of patients that receive an induction agent at the time of kidney transplantation receive rATG. Induction doses have ranged from 1–6 mg/kg/dose over 1–10 days with a more typical regimen of 1.5 mg/kg for 3–5 days [3–11] with a cumulative target of 4.5–10 mg/kg.

In animal models, higher initial doses of shorter duration approximating a human-equivalent dose of 6 mg/kg were associated with more peripheral and central lymphocyte depletion and better allograft survival [12]. In humans, total doses of 5.7 mg/kg on average given as 1.5 mg/kg per day have been shown to produce similar outcomes in high risk recipients who received an average of 10.3 mg/kg [9]. Based on these models the optimal induction dose is felt to approximate 6 mg/kg [4, 7–10, 12].

Higher doses and prolonged duration of induction agents are thought to be associated with an increased risk of infection and the potential development of malignancy, while low doses, <3 mg/kg, may not effectively prevent acute rejection [11]. Thus, the optimal dose and duration of rATG have not been clearly defined. Therefore, the purpose of this study was to evaluate the safety and efficacy of two dosing strategies of rATG, 1.5 mg/kg given for 4 days versus 2 mg/kg given for 3 days.

## 2. Materials and Methods

This retrospective, single center, sequentially designed cohort study evaluated adult renal transplant recipients receiving rATG at Washington University/Barnes-Jewish Hospital. Those transplanted between October 2005 and March 2006 who received 1.5 mg/kg of rATG for 4 days were compared to those transplanted after April 2006 who received 2 mg/kg of rATG for 3 days. The study was approved by the Washington University Human Research Protection Office. 

### 2.1. Patient Inclusion and Exclusion Criteria

All adult renal transplant recipients were considered for enrollment. Patients were excluded if their immunosuppression deviated from protocol for the following reasons; those with a six–antigen-matched living donor who do not receive induction therapy at our institution, those with serologic evidence of hepatitis B virus (HBV), hepatitis C virus (HCV), or multiple organ transplant who may not receive full dose induction, and patients enrolled in an investigational study.

### 2.2. Immunosuppression

Patients received quadruple sequential immunosuppression consisting of induction with rATG, followed by triple maintenance immunosuppression with tacrolimus, azathioprine, or mycophenolic acid, and prednisone. 

The first dose of intravenous rATG was given over twelve hours beginning before reperfusion. Subsequent doses were given over six hours and withheld if the platelet count dropped below 50,000 per mm^3^ or the white blood cell (WBC) count dropped below 2,000 per mm^3^. If the platelet count was between 50,000–75,000 per mm^3^ or the WBC count was between 2,000–3,000 per mm^3^ the rATG dose was halved. Patients in the 1.5 mg/kg group received 1.5 mg/kg of rATG (based on total body weight with a maximum dose 150 mg per day) for 4 days and were compared to patients that received 2 mg/kg (based on total body weight with a maximum dose 200 mg per day) for 3 days. If a dose was held for any reason it was not given the next day. Premedications such as dipenydramine or acetaminophen were not routinely given. Corticosteroids were administered daily.

Tacrolimus was initiated when patients achieved a brisk diuresis (but no later than 3 days postoperatively) at a dose of 0.1 mg/kg per day divided in two doses. Tacrolimus trough levels were targeted at 5–10 ng/mL by microparticle enzyme immunoassay (MEIA) (IMx, Abbott Laboratories, Abbott Park, IL). Methylprednisolone was administered intraoperatively (7 mg/kg) to a maximum of 500 mg, followed by prednisone 1 mg/kg/day to a maximum of 80 mg orally on days in which rATG was administered and then tapered over 5 weeks to 5 mg/day. Mycophenolate mofetil at a dose of 500–1000 mg or enteric coated mycophenolic acid sodium at a dose of 360–720 mg was administered orally twice daily.

### 2.3. Antimicrobial Prophylaxis

Patients received nystatin suspension for three months as fungal prophylaxis and sulfamethoxazole/ trimethoprim double strength daily for *Pneumocystis jiroveci* and bacterial prophylaxis. For patients allergic to sulfa, dapsone 50 mg daily was substituted. For viral prophylaxis, when either the donor or recipient had serologic evidence of prior exposure to cytomegalovirus (CMV), oral valganciclovir was given, 450 mg daily for 3–12 months based on CMV risk status. Cytomegalovirus-seronegative recipients of a CMV-seronegative donor received acyclovir 200 mg orally twice daily for 3 months posttransplant for Herpes simplex prophylaxis.

### 2.4. Outcomes and End Points

The primary endpoint of this study was the rate of acute rejection. Secondary analysis included the time to and rate of serious infections, patient survival, graft survival, acute rejection, incidence of malignancy including posttransplant lymphoproliferative disorder, complete blood counts, and renal function as assessed by serum creatinine and estimated glomerular filtration rate. Rabbit antithymocyte globulin drug costs were estimated based on average wholesale price in 2009 [13].

### 2.5. Definitions

Serious infection was defined as an infection prolonging the initial hospital stay or requiring hospital admission. Cytomegalovirus infection and disease were defined as previously described [14, 15]. Acute rejection episodes were determined by the presence of clinical signs, including a rise in serum creatinine and were confirmed in all cases by biopsy as defined by the Banff '97 criteria [16]. Glomerular filtration rate was estimated by the abbreviated modification of diet in renal disease (MDRD) formula [17]. Antibody results (ELISA or MHC class I or II antibodies) are expressed as the number and percentage of patients with any class I or II antibodies.

### 2.6. Database and Patient Followup

Patients at our center are followed with a minimum of monthly labs and yearly clinic visits. Patients are tracked at least yearly and their clinical status is reported to UNOS. Our center uses an electronic medical record system, Organ Transplant Tracking Record (OTTR—HKS Medical Information Systems, Omaha, NE). In addition, a research nurse monitors and records all pertinent data from the medical records in a database for research purposes.

### 2.7. Sample Size

The sample size was determined based on an alpha of 0.05 and a power of 0.80, to find a 25% difference in acute rejection. To achieve this at least 59 patients needed to be analyzed in each group.

### 2.8. Statistics

Incidence of infection, acute rejection, graft loss, and death were calculated using survival analysis techniques. Univariate analysis was performed by the Student's *t*-test for continuous variables and the Chi-squared test for categorical variables. All statistical tests were two-tailed and a *P* value of <0.05 was considered significant.

## 3. Results

Between October 2005 and September 2006, 150 kidney transplants were performed. Patients were excluded because of multiple organ transplant (*n* = 7), positive HCV serology (*n* = 1), no induction due to a six-antigen-matched live donor (*n* = 2), and participation in an investigational study (*n* = 22). The average time of followup was 2.3 ± 0.1 years in the 1.5 mg/kg group and 1.9 ± 0.1 in the 2 mg/kg group.

### 3.1. Recipient and Donor Characteristics

There were no differences in baseline recipient or donor characteristics Table 1. Overall, the mean recipient age was 51 years with approximately 1/3 of patients over the age of 60 years. Eighteen percent of the patients were black. Most recipients were male, not sensitized, and received a first deceased donor allograft.

### 3.2. Rejection and Graft Function

At two-years after transplantation rejection-free survival was 95% in both groups, *P* = .983. At last followup, 3 patients in the 1.5 mg/kg group and 3 patients in the 2 mg/kg group had suffered an acute rejection Table 2. Grade I rejections were most common in both groups, although one patient in the 2 mg/kg group suffered a grade III rejection. Most of the rejections occurred within the first year after transplantation, although one 1.5 mg/kg patient suffered a late rejection at 540 days after transplantation. 

Serum creatinine was similar in the 1.5 mg/kg group and the 2 mg/kg group at baseline (7.6 ± 3.3 mg/dL versus 8.4 ± 3.6 mg/dL, *P* = 0.207), 6 months (1.4 ± 0.4 mg/dL versus 1.3 ± 0.4 mg/dL, *P* = .383), 12 months after transplantation (1.4 ± 0.5 mg/dL versus 1.4 ± 0.5 mg/dL, *P* = 0.748), and at last followup (1.6 ± 1.3 mg/dL versus 1.6 ± 0.9 mg/dL, *P* = .898). The estimated glomerular filtration rate [16] was similar in the 1.5 mg/kg group and the 2 mg/kg group at baseline (10 ± 5 mL/min versus 10 ± 7 mL/min, *P* = .766), 6 months (67 ± 29 mL/min versus 69 ± 22 mL/min, *P* = .646), at 12 months after transplantation (68 ± 37 mL/min versus 67 ± 23 mL/min, *P* = .867), and last followup (65 ± 39 mL/min versus 68 ± 23 mL/min, *P* = .642).

### 3.3. Infection

**Figure 1:**
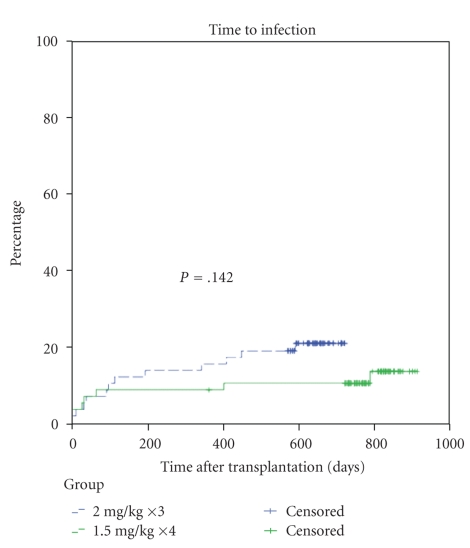
Incidence of infection. The two-year incidence of infection was similar between the groups, 12% in the 1.5 mg/kg group and 20% in the 2 mg/kg group, *P* = .142.

The two-year incidence of serious infection was similar between the groups, 12% in the 1.5 mg/kg group and 20% in the 2 mg/kg group, *P* = 0.142 Figure 1. Eight patients had infections in the 1.5 mg/kg group that consisted of 6 bacterial infections and 2 viral infections Table 2. Thirteen patients had infections in the 2 mg/kg group; 8 were bacterial, 3 viral, and 1 fungal. There incidence of CMV viremia was similar (1 (2%) in the 1.5 mg/kg group and 2 (3%) in the 2 mg/kg group). Blood was the most common site for infection in both groups. There were no cases of BK nephropathy during this time period. 

### 3.4. Survival

Two-year patient survival was similar between the groups, 97% in the 1.5 mg/kg group versus 100% in the 2 mg/kg group, *P* = .317. At last followup, two patients in the 1.5 mg/kg group expired: a 67-year-old from myocardial infarction at two years after transplantation and a 30-year-old recipient of a kidney after lung transplant from adenocarcinoma of unknown primary origin. All patients were alive in the 2 mg/kg group. Two-year graft survival was similar between the groups, 92% in the 1.5 mg/kg group versus 95% in the 2 mg/kg group, *P* = .766. Five patients lost their allograft in the 1.5 mg/kg group: 1 from early thrombosis, 1 from early rejection, 1 at 403 days (a patient who received an ECD kidney complicated by DGF, acute rejection, CMV viremia, BK viremia, and listeria) and 2 died with a functioning graft. Three patients in the 2 mg/kg group lost their allografts: 1 due to early rejection, 1 from metabolic complications associated with a persistent eating disorder, and 1 due to refractory plasmacytic rejection in a patient with HCV.

### 3.5. Safety and Malignancy

**Figure 2:**
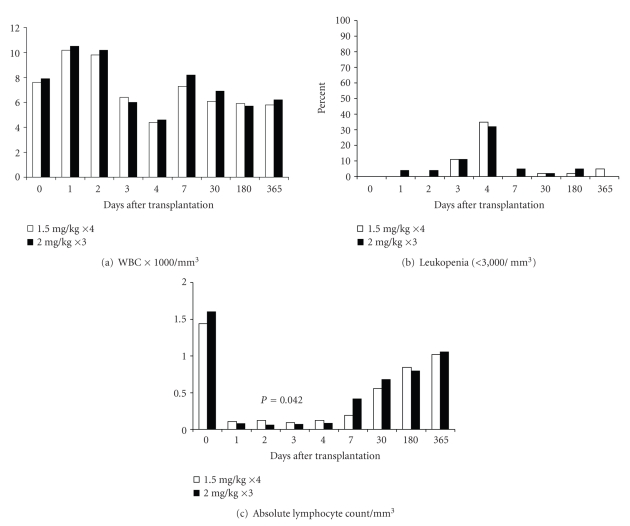
Figures 2(a), 2(b), and 2(c). White blood cell counts and incidence of leucopenia. White blood cell counts and the percentage of patients with leucopenia were similar in both groups throughout one year after transplantation. The absolute lymphocyte count was statistically lower in the 2 mg/kg arm on postoperative day 2 (*P* = .42) but similar throughout the rest of the study period.

**Figure 3:**
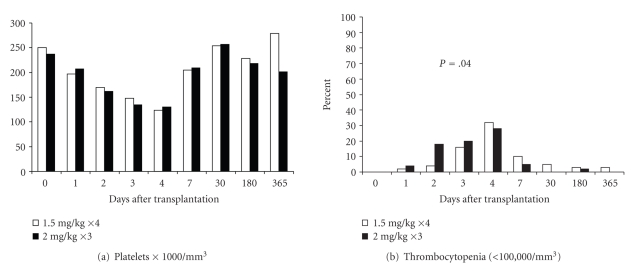
Figures 3(a), and 3(b). The mean platelet counts were similar throughout the study period, while the percentage of patients with thrombocytopenia was higher in the 2 mg/kg arm on postoperative day 2. At 2 days postoperatively, 4% of the 1.5 mg/kg group's patients had thrombocytopenia while 28% of the 2 mg/kg group had thrombocytopenia, *P* = .04.

White blood cell counts and the percentage of patients with leucopenia were similar in both groups throughout one-year after transplantation Figures 2(a) and 2(b). The absolute lymphocyte count was statistically lower in the 2 mg/kg arm on postoperative day 2, *P* = .042 Figure 2(c). The mean platelet counts were similar throughout the study period, while the percentage of patients with thrombocytopenia was higher in the 2 mg/kg arm on postoperative day 2, (4%—1.5 mg/kg for 4 days versus 28%—2 mg/kg for 3 days, *P* = .04) Figures 3(a) and 3(b). No patients received colony stimulating factors and serum sickness did not occur.

Malignancy occurred in 3 patients in the 1.5 mg/kg group (1 squamous cell cancer of the skin, 1 basal cell cancer of the right temple, and 1 adenocarcinoma) and 1 patient in the 2 mg/kg group (esophageal cancer). There were no cases of posttransplant lymphoproliferative disorder. The average length of stay for the transplant admission tended to be longer in the 1.5 mg/kg for 4 days group compared to the 2 mg/kg for 3 days group (6.0 ± 3.7 days versus 5.1 ± 1.9 days, *P* = .104).

### 3.6. Immunosuppression

The mean total dose of rATG in the 1.5 mg/kg group was 474 ± 132 mg given over 3.7 ± 0.7 days (cumulative dose 5.7 ± 1.6 mg/kg) and the mean dose of rATG in the 2 mg/kg group was 450 ± 119 mg given over 3.0 ± 0.5 days (cumulative dose 5.6 ± 1.3 mg/kg). Twelve patients (20%) in the 1.5 mg/kg for 4 days arm did not receive four doses of rATG (11 received fewer doses (19%) and 1 received 5 doses). Nine patients (15%) in the 2 mg/kg × 3 days arm did not receive 3 doses of rATG (6 received fewer doses (10%) and 3 received 4 doses (5%)). The most common reasons for a receiving fewer doses of rATG were thrombocytopenia and leucopenia in both arms. In the 1.5 mg/kg for 4 days arm, 3 repeat transplant recipients received only 2-3 doses of 1.5 mg/kg because of concerns over excessive immunosuppression. One patient with prolonged delayed graft function received an additional dose of rATG. In the 2 mg/kg for 3 days arm, 3 patients received 4 doses of 1.5 mg/kg due to older age and debility.

### 3.7. Pharmacoeconomic

Average wholesale price (AWP) for rATG was $ 610.00/ 25 mg vial [13]. The mean cost of rATG in the 1.52 mg/kg group was $11,569 ± 3,239 and in the 2 mg/kg group was $10,649 ± 3,178 (*P* = .122), demonstrating a cost savings of $920.

## 4. Discussion

Studies conducted using rATG for induction have evaluated the efficacy and safety of a 7-day course of therapy dosed at 1.5 mg/kg/day [3] as well as a shorter, lower dose regimens (3–6 mg/kg total dose) administered over 3 days [4, 7]. Dosing regimens have ranged from total cumulative dose of 3–10.5 mg/kg [2, 10]. Innovative dosing regimens have been described, including one-time high dose administration of 5 mg/kg [6, 10], intermittent dosing of rATG based on CD3^+^ lymphocyte counts [5], and alternate day therapy [7, 10]. Various timing, dosage and duration strategies documented for rATG as induction therapy are summarized in Table 3. Although allograft outcomes appear to be similar among regimens, one trial demonstrated improved early renal function (between postoperative days 1 through 4) in renal transplant recipients that received a one time dose rATG dose of 6 mg/kg versus 1.5 mg/kg every other day for 4 doses [10]. The authors speculated that rATG may induce a dose-dependent reduction in reperfusion injury.

Cumulative doses of rATG less than 7.5 mg/kg appear to be effective in high risk renal transplant recipients [5, 8, 9]. Wong et al. published a recent, retrospective cohort study that targeted rATG doses of 1.5 mg/kg given for 7–10 days. Actual doses were significantly less than anticipated and lower cumulative doses of rATG (less than 7.5 mg/kg) were as safe and effective as higher cumulative doses of rATG (greater than 7.5 mg/kg). Acceptable rates of rejection and survival were also demonstrated in a prospective, single group trial of kidney and kidney-pancreas recipients that received 1.5 mg/kg of rATG doses based on CD3+ lymphocyte counts (mean total cumulative dose 4.2 mg/kg) [7].

The present sequentially designed, retrospective cohort study of 1.5 mg/kg of rATG for 4 days (*n* = 59) versus 2 mg/kg of rATG for 3 days (*n* = 59) demonstrates that both dosing strategies are equally efficacious. Furthermore, a higher daily dose (2 mg/kg with a maximum dose of 200 mg) than the recommended (1.5 mg/kg with a maximum dose of 150 mg [1]) did not result in an increased risk of infection or cancer and was associated with lower total mean dose of rATG. 

The benefits of a 2 mg/kg for 3-day dosing regimen of rATG may include better use of healthcare resources with opportunities for a lower dose and shorter length of the initial transplant admission. In this study, the 2 mg/kg group spent one less day in the hospital when compared to the 1.5 mg/kg group. Larger studies powered to detect a difference in hospital stay are needed to determine the full impact of alternative dosing strategy on length of stay.

This study was performed as part of our ongoing quality control measures to assess the impact of new regimens. The major limitation of the study is that it was retrospectively conducted. A study with larger patient numbers may be necessary to demonstrate a difference in cost and length of stay. Additionally, longer followup is warranted to fully study the impact of rATG on malignancy. The external validity of the study should also be considered. Most of the patients in this study were Caucasian, first time transplant recipients, and 43% of the recipients received live donor allografts.

Shorter, more intense dosing of rATG is safe and effective. This cohort study comparing rATG 1.5 mg/kg for 4 days versus 2 mg/kg for 3 days demonstrates that both dosing strategies are equally tolerated while allowing a low rate of acute rejection. This alternate dosing strategy may allow for shorter lengths of stay and lower mean doses, thus cost savings.

## Figures and Tables

**Table 1:** Recipient and Donor Characteristics.

Recipient Characteristics	1.5 mg/kg x 4 days (*n* = 59)	2 mg/kg x 3 days (*n* = 59)	*P* value
Age, yrs	52 ± 13	49 ± 13	.136
% of Patients >60 yrs, Number (%)	22 (37%)	16 (27%)	.237
Race, Number (%)			.490
White	46 (78%)	44 (75%)	
Black	12 (20%)	11 (19%)	
Gender, Number (%)			.456
Female	23 (39%)	27 (46%)	
Weight (kg)	84 ± 18	79 ± 21	.215
Body Mass Index (kg/m^2^)	28 ± 5	27 ± 7	.566
Transplant Type, Number (%)			.353
Deceased Donor	31 (53%)	36 (61%)	.559
Expanded Criteria Donor	9 (15%)	1 (2%)	.008
Pretransplant Dialysis	43 (73%)	44 (75%)	.834
Transplant Number, Number (%)			
First	57 (97%)	58 (98%)	.559
Cause of Renal Disease, Number (%)			.572
Hypertension	22 (37%)	14 (24%)	
Diabetes	10 (17%)	10 (17%)	
Polycystic Kidney Disease	10 (17%)	9 (15%)	
Glomerulonephritis	10 (13%)	14 (24%)	
Lupus	1 (2%)	3 (5%)	
Other	6 (10%)	9 (15%)	
Donor Age	39 ± 14	37 ± 11	.412
CMV Serostatus Donor/Recipient, Number (%)			.381
Negative/negative	8 (14%)	10 (17%)	
Positive/negative	16 (27%)	9 (15%)	
Negative/positive	17 (29%)	16 (27%)	
Positive/positive	18 (31%)	24 (41%)	
Class I antibody, Number (%)	6 (10%)	14 (24%)	.050
Class II antibody, Number (%)	9 (15%)	7 (12%)	.590
Cold Ischemia Time, (hrs)	11 ± 5	13 ± 6	.110
Delayed Graft Function, Number (%)	4 (7%)	5 (8%)	.729

Mean ± SD.

Abbreviations: ESRD: end stage renal disease, CMV: cytomegalovirus.

**Table 2:** Outcomes.

	1.5 mg/kg × 4 days (*n* = 59)	2 mg/kg × 3 days (*n* = 59)	*P* value
Patient Survival, #(%)	57 (97%)	59 (100%)	.317
Graft Survival, #(%)	54 (92%)	56 (95%)	.766
Freedom from Rejection, #(%)	56 (95%)	56 (95%)	.983
Grade of Rejection, #(%)			
Grade I	3	2	
Grade II	0	0	
Grade III	0	1	
Infection #(%)* ^ ^	7 (12%)	12 (20%)	.142
Viral	2	3	
Fungal	0	1	
Bacterial	6	8	
Site			
Blood	5	5	
Urine	1	1	
Skin and Soft Tissue	1	3	
Lung	0	1	
Other/ Unknown	1	2	
PTLD, #(%)	0 (0%)	0 (0%)	
Tumor, #(%)	3 (5%)	1 (2%)	.309
CMV Infection, #(%)	1 (2%)	2 (3%)	.154
Length of Stay (days)	6.0 ± 3.7	5.1 ± 1.9	.104
Serum Creatinine (mg/dL)			
Baseline	7.6 ± 3.3	8.4 ± 3.6	.207
6 months	1.4 ± 0.4	1.3 ± 0.4	.383
12 months	1.4 ± 0.5	1.4 ± 0.5	.748
Last followup	1.6 ± 1.3	1.6 ± 0.9	.898
GFR (mg/mL)			
Baseline	10 ± 5	10 ± 7	.766
6 months	67 ± 29	69 ± 22	.646
12 months	68 ± 37	67 ± 23	.867
Last followup	65 ± 39	68 ± 23	.642
Total Dose (mg)	474 ± 132	450 ± 119	.308
rATG Doses (#)	3.7 ± 0.7	3.0 ± 0.5	

Mean ± SD.

Abbreviations: PTLD, posttransplant lymphoproliferative disorder, CMV, cytomegalovirus, GFR, glomerular filtration rate.

*One patient had a bacterial and viral infection.

**Table 3:** Various rATG dosing regimens.

Author, Center	Study Design	Organ	rATG Regimen	Control
Brennan, Barnes-Jewish, Wash U [3]	Prospective, Randomized, Double-blinded	K	1.5 mg/kg × 7days, total 10.5 mg/kg (*n* = 48)	Atgam 15 mg/kg × 7d(*n* = 24)
Agha, Barnes-Jewish, Wash U [4]	Prospective, Nonrandomized	K	3.0 mg/kg/day, then 1.5 mg/kg × 2 days, total 6 mg/kg (*n* = 40)	1.5 mg/kg × 7d, total 10.5 mg/kg(*n* = 48)
Peddi, U of Cincinnati [5]	Prospective, Single-group	High risk K, KP	1.5 mg/kg, Intermittent dosing, based on CD3^+^monitoring, mean total 4.2 mg/kg (*n* = 41)	Uncontrolled
Starzl, Pittsburg[6]	Prospective, Single-group	K, P, L	~5 mg/kg × 1 dose, total 5 mg/kg (*n* = 82)	Uncontrolled
Stratta, Wake Forest [7]	Retrospective, Single group	P	1.5 mg/kg, Alternate-day therapy until calcineurin inhibitor levels are achieved, mean total 5.4 mg/kg (*n* = 55)	Uncontrolled
Wong, Massachusetts General Hospital [8]	Prospective	K	1.0 mg/kg × 3 day, total 3 mg/kg (*n* = 7)	1.5 mg/kg × 3 d, total 4.5 mg/kg (*n* = 9)
Gurk, U of Maryland [9]	Retrospective cohort	High risk K	Target 1.5 mg/kg × 7-10day< 7.5 mg/kg (*n* = 33)	>7.5 mg/ kg (*n* = 64)
Stevens, U of Nebraska [10]	Prospective, Randomized	K	6 mg/kg × 1 dose (*n* = 70)	1.5 mg/kg qod × 4 doses, total dose 6 mg/kg (*n* = 72)

Abbreviations: K, kidney, P, pancreas, L, liver, KP, kidney pancreas.
